# 

**Published:** 1882-08

**Authors:** 


### Pamphlets.

---

Infant Feeding and Infant Foods. The anniversary address delivered before the New York State Medical Society, by Abraham Jacobi, M D. Reprint from the *Medical News.*

Static Electricity as a Therapeutic Agent. By James Knight, M.D.

On Genital Renovation by Kolpostenoteny and Kolpoecpetasis in Urinary and Fecal Fistules. By Nathan Bozeman, M.D., New York. Reprint from *Gynecological Transactions* 1882.

Plastic Splints in Surgery. By Samuel N. Nelson, A.B., M.D. Boston. Reprint from *Annals of Anatomy and Surgery.*

Double Irrigation and Drainage Tubes. Uterine Dilatation by Elastic Force. The Cure of Hernia by the Antiseptic Use of Animal Ligature. By Henry O. Marcy, A.M., M.D. Boston. Reprint from *Transactions of the International Medical Congress for* 1881.

Report on Surgery. By W. O. Roberts, M.D. Reprint from the *American Practitioner.*

Second Annual Report of the Astronomer in Charge of the Horological and Thermometrical Bureaus in the Observatory of Yale College. 1881–2. By Leonard Waldo. New Haven.

Address Delivered by the President of the American Institute of Homœopathy, William L. Breyfogle, M.D., at its Thirty-fifth Annual Session, held at Indianapolis, Indiana, June 13 to 17. 1882.

The American Bulletin. Issued occasionally by the New York Committee of the International Federation to Promote the Abolition of State-Regulated Vice.

